# Involvement of N- and C-terminal region of recombinant cervid prion protein in its reactivity to CWD and atypical BSE prions in real-time quaking-induced conversion reaction in the presence of high concentrations of tissue homogenates

**DOI:** 10.1080/19336896.2020.1858694

**Published:** 2020-12-19

**Authors:** Akio Suzuki, Kazuhei Sawada, Takeshi Yamasaki, Nathaniel D Denkers, Candace K Mathiason, Edward A Hoover, Motohiro Horiuchi

**Affiliations:** a Laboratory of Veterinary Hygiene, Graduate School of Infectious Diseases, Hokkaido University, Kita-ku, Sapporo, Japan; b Prion Research Center, Department of Microbiology, Immunology, and Pathology, College of Veterinary Medicine and Biomedical Sciences, Colorado State University, Fort Collins, Colorado, USA; c Global Station for Zoonosis Control. Global Institute for Collaborative Research and Education, Hokkaido University, Kita-ku, Sapporo, Japan

**Keywords:** bovine spongiform encephalopathy, cervid prion protein, chronic wasting disease, prion, real-time quaking-induced conversion

## Abstract

The real-time quaking-induced conversion (RT-QuIC) reaction is a sensitive and specific method for detecting prions. However, inhibitory factors present in tissue homogenates can easily interfere with this reaction. To identify the RT-QuIC condition under which low levels of chronic wasting disease (CWD) and bovine spongiform encephalopathy (BSE) prions can be detected in the presence of high concentrations of brain tissue homogenates, reactivities of various recombinant prion proteins (rPrPs) were tested. Among the tested rPrPs, recombinant cervid PrP (rCerPrP) showed a unique reactivity: the reactivity of rCerPrP to CWD and atypical BSE prions was not highly affected by high concentrations of normal brain homogenates. The unique reactivity of rCerPrP disappeared when the N-terminal region (aa 25–93) was truncated. Replacement of aa 23–149 of mouse (Mo) PrP with the corresponding region of CerPrP partially restored the unique reactivity of rCerPrP in RT-QuIC. Replacement of the extreme C-terminal region of MoPrP aa 219–231 to the corresponding region of CerPrP partially conferred the unique reactivity of rCerPrP to rMoPrP, suggesting the involvement of both N- and C-terminal regions. Additionally, rCer^N^–Mo–Cer^C^PrP, a chimeric PrP comprising CerPrP aa 25–153, MoPrP aa 150–218, and CerPrP aa 223–233, showed an additive effect of the N- and C-terminal regions. These results provide a mechanistic implication for detecting CWD and atypical BSE prions using rCerPrP and are useful for further improvements of RT-QuIC.

## Introduction

Prion diseases are fatal neurodegenerative disorders including Creutzfeldt-Jakob disease (CJD), Gerstmann-Straussler-Scheinker syndrome (GSS), and fatal insomnia in humans, scrapie in sheep and goats, chronic wasting disease (CWD) in cervids and bovine spongiform encephalopathy (BSE) [1]. Prions, which are the causative agents of prion diseases, are mainly composed of an abnormal isoform of the prion protein (PrP^Sc^) that are generated from host-encoded cellular isoform of the prion protein (PrP^C^). Binding of PrP^C^ to PrP^Sc^ induces a conformational conversion from the α-helix-rich PrP^C^ to the β-structure-rich PrP^Sc^. Accumulation of PrP^Sc^ in the central nervous system is the most characteristic feature of these diseases.

CWD was first identified in 1967 in a group of captive mule deer and was classified as a TSE in 1980. To date, CWD-affected cervids have been found in the US, Canada, South Korea [2,3], and Scandinavian countries [4,5]. Different from other prions, infectious CWD prions have also been detected in secreted body fluids and excretions such as saliva, urine, and faeces [6,7]. Although CWD did not transmit to mice expressing human PrP [8,9], the zoonotic potential of CWD cannot be ignored, as CWD prions are known as experimentally transmissible to several animals including the squirrel monkey [10], and prion properties change during interspecies transmission [11–14].

The classical BSE (C-BSE) was recognized in the United Kingdom in 1986 [15], and since then has spread globally. C-BSE is known as a cause of zoonotic prion diseases as it is transmitted to human via BSE-contaminated products, which has caused the emergence of variant CJD [16]. C-BSE is now under control due to worldwide implementation of control measures such as feed bans. Two atypical BSEs were identified in 2004, and they were classified as H- and L-BSE based on the higher and lower apparent molecular weights of un-glycosylated PrP^Sc^ observed in immunoblotting [17]. Atypical BSE cases were mainly disclosed in cattle over the age of 8 years old and are also found worldwide, including in Brazil wherein no C-BSE case has been reported, suggesting that atypical BSEs may be sporadic diseases that occur in aged cattle, similar to sporadic CJD in humans [18]. L-BSE is known to be experimentally transmissible to primates and mice expressing human PrP [19–21], whereas the zoonotic potential of H-BSE has not yet been fully elucidated. Since low levels of prion infectivity and seeding activity of atypical BSE prions were detected in skeletal and intercostalis muscles [22,23] and in tonsillar tissue [24] from cattle affected with atypical BSEs, a potential risk of human transmission via food consumption cannot be ignored.

To disclose the potential presence of CWD and atypical BSEs, highly sensitive and accurate methods are required for detecting low levels of prions in tissue homogenates or body fluids. Bioassays using appropriate gene-modified mice could be sensitive enough to detect low levels of prions [24,25]; however, bioassays require extremely long experimental time periods to detect low levels of infection. Highly sensitive *in vitro* methods such as protein misfolding cyclic amplification (PMCA) [26] and quaking-induced conversion [27] have been reported to successfully detect the low level of prions. Real-time quaking-induced conversion (RT-QuIC), which detects the amyloid seeding activity of PrP^Sc^, is known as a specific and highly sensitive assay capable of detecting low levels of prions [28–31]. However, the inhibitory factors in tissue homogenates and body fluids can interfere with the reaction [31–33], which then hampers detection of low level prions in the presence of high concentrations of tissue homogenates. In the recent study, we showed that recombinant cervid PrP (rCerPrP) is useful for the detection of atypical BSE prions from tissues of cattle affected with atypical BSEs [22]. In the present study, we show that the RT-QuIC reaction using full-length rCerPrP is less affected by a high concentration of brain tissue homogenates when detecting CWD and atypical BSE prions. We also analysed the region(s) responsible for the unique properties of rCerPrP in the reaction.

## Results

### Detection of CWD prions in the presence of high concentration of brain tissue homogenates

**Figure 1.**
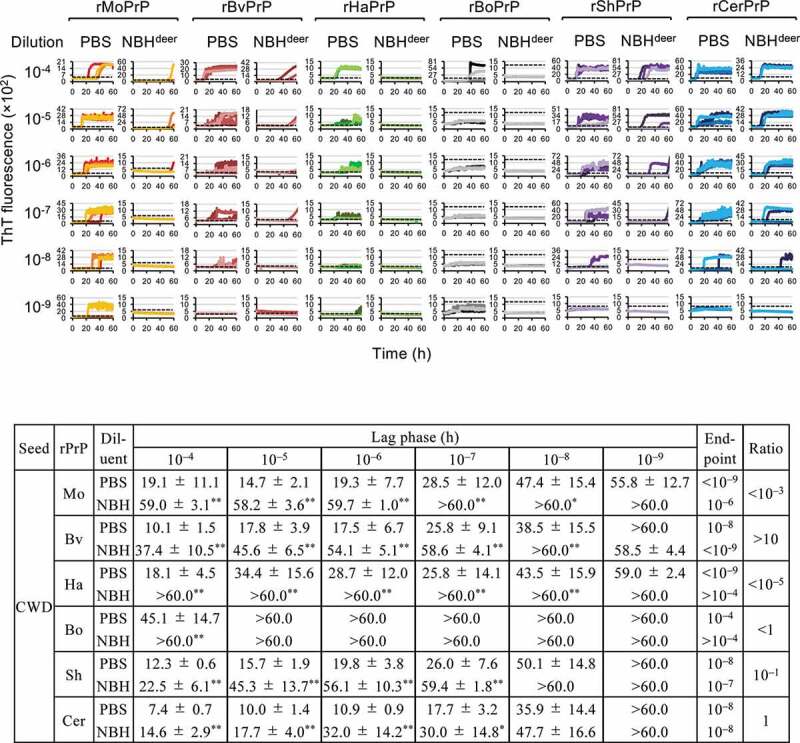
Reactivity of rPrPs to CWD prions in RT-QuIC in the presence and absence of normal brain homogenates. Representative line graphs for the detection of CWD prions serially diluted with PBS (left column) or 2% brain homogenates in PBS of CWD-negative white-tail deer (normal brain homogenate, NBH) (final concentration of NBH in the reaction mixture was 0.1%) (right column). Detections performed using rMoPrP (red to yellow), rBvPrP (brown), rHaPrP (green), rBoPrP (black), rShPrP (purple), and rCerPrP (blue) are shown. ThT intensities from triplicate wells were plotted against reaction time using different brightness or colours. Dotted lines indicate the thresholds of reaction calculated from the mean ThT fluorescence intensity plus 5 × SD of negative control wells (without brain homogenates). Lag phases (mean ± SD), endpoints, and ratios of the endpoints [Ratio, endpoint (PBS)/endpoint (NBH)] are summarized in the table. *: p < 0.05, **: p < 0.01 by welch t-test

We analysed the reactivity of six rPrPs to CWD prions in the presence and absence of 0.1% deer NBH (Figure 1). The endpoints of the reactions using the five rPrPs (rMoPrP, rBvPrP, rHaPrP, rShPrP, and rCerPrP) were from 10^−8^ to <10^−9^, in the absence of NBH (Figure 1, PBS); however, the lag phases were varied with the rPrPs. Among the five rPrPs, the lag phases using rCerPrP were the shortest at each seed dilution. rBoPrP was ineffective in detecting CWD prions, as the endpoint of the reaction was 10^−4^ (Figure 1). Interestingly, the reactions of rHaPrP, rMoPrP, and rShPrP were affected by 0.1% deer NBH: the rHaPrP reaction was completely inhibited, and the endpoints of reactions using rMoPrP and rShPrP worsened by 3 and 1 logs, respectively (Figure 1). Additionally, lag phases of the reactions with each rPrP were significantly prolonged: the lag phases of the reactions using rMoPrP, rBvPrP, and rShPrP were prolonged by 43.5, 27.8, and 29.6 h, respectively, at 10^−5^ seed dilution in the presence of 0.1% deer NBH (Figure 1). In contrast, the rCerPrP reaction was not severely affected by 0.1% deer NBH; the endpoint was unchanged, and the lag phase at 10^−5^ seed dilution was only prolonged by 7.7 h (Figure 1).

### Detection of atypical and classical BSE prions in the presence of high concentration of brain tissue homogenates

We analysed the reactivity of six rPrPs to atypical BSE (H- and L-BSE) and classical BSE (C-BSE) in RT-QuIC. Although rBoPrP showed longer lag phases than the other rPrPs, all rPrPs showed similar endpoints for detecting H-BSE prions in the absence of NBH (10^−8^ or <10^−9^, Table 1). rBoPrP and rHaPrP seemed to be less effective than the other four rPrPs in detecting L-BSE prions: endpoints of reactions using the former rPrPs (10^−6^ and 10^−7^) were lower than the latter rPrPs (10^−8^ or <10^−9^). A similar tendency was observed for the detection of C-BSE prions: rBoPrP and rHaPrP (endpoints: 10^−4^ and 10^−5^) appeared to be less effective than the four other rPrPs (endpoints: 10^−6^). Although no difference was observed in the detection endpoints of H-BSE prions among rMoPrP, rBvPrP, rShPrP, and rCerPrP, rCerPrP was able to detect H-BSE prions with shorter lag phases than the other rPrPs (Table 1). Interestingly, 0.1% cattle NBH completely interfered with the detection of H-BSE prions with rBvPrP and rHaPrP, detection of L-BSE prions with rMoPrP, rBvPrP, rHaPrP, and rBoPrP, and detection of C-BSE prions with the five rPrPs except for rCerPrP (Table 1). Notably, detection of atypical BSE prions with rCerPrP was not severely affected by 0.1% cattle NBH: endpoints of the reaction were unchanged and lag phases were prolonged at some seed dilutions, e.g, at 10^−5^ to 10^−7^ for H-BSE, and at 10^−4^ for L-BSE, but the differences were not that large (<10 h except at 10^−7^ seed dilution for H-BSE, Table 1). These results suggest that rCerPrP is a good substrate for the detection of atypical BSE as well as CWD prions in the presence of high concentrations of brain tissue homogenates. Moreover, detection of C-BSE prions was affected by 0.1% cattle NBH, which the endpoint of the reaction worsened by 2 logs and the lag phases were significantly prolonged (Table 1). Also, 0.1% cattle NBH did not interfere with the detection of atypical BSE prions but interfered with detection of C-BSE prions, suggesting that RT-QuIC using rCerPrP can discriminate atypical BSE from C-BSE prions.

**Table 1.** Reactivity of rPrPs to atypical and classical BSE prions in RT-QuIC

Seed	rPrP	Diluent ^(a)^	Lag phase (h)^(^^b)^						End-point	Ratio^(^^c)^
			10^–4^	10^–5^	10^–6^	10^–7^	10^–8^	10^–9^		
H-BSE	Mo	PBS	21.2 ± 6.6	29.0 ± 12.3	28.8 ± 3.5	35.0 ± 13.4	49.1 ± 10.4	57.2 ± 8.3	<10^–9^	<10^–2^
		NBH	50.4 ± 9.9**	56.0 ± 5.4**	59.2 ± 2.4**	59.5 ± 1.4**	>60.0**	>60.0	10^–7^	
	Bv	PBS	10.6 ± 1.5	21.9 ± 7.7	21.9 ± 3.8	27.6 ± 6.2	35.4 ± 10.5	55.5 ± 9.2	<10^–9^	<10^–5^
		NBH	>60.0**	>60.0**	>60.0**	>60.0**	>60.0**	>60.0	>10^–4^	
	Ha	PBS	11.2 ± 1.1	26.3 ± 7.7	28.8 ± 8.5	43.5 ± 16.6	54.1 ± 8.8	>60.0	10^–8^	<10^–4^
		NBH	> 60.0**	> 60.0**	> 60.0**	> 60.0**	> 60.0	> 60.0	>10^–4^	
	Bo	PBS	34.1 ± 3.0	41.2 ± 3.2	46.8 ± 7.4	56.9 ± 4.6	59.2 ± 2.3	58.7 ± 4.0	<10^–9^	<10^–4^
		NBH	59.0 ± 2.6**	59.7 ± 0.8**	>60.0**	>60.0	>60.0	>60.0	10^–5^	
	Sh	PBS	12.0 ± 1.1	14.5 ± 1.5	18.7 ± 3.1	19.9 ± 2.2	31.0 ± 12.6	56.2 ± 10.1	<10^–9^	1
		NBH	13.3 ± 1.8	14.4 ± 1.4	16.4 ± 0.9	19.2 ± 2.9	24.6 ± 7.9	52.3 ± 12.6	<10^–9^	
	Cer	PBS	10.8 ± 1.9	10.9 ± 1.6	14.7 ± 2.8	16.9 ± 4.1	30.2 ± 7.9	58.2 ± 3.4	<10^–9^	1
		NBH	20.4 ± 15.6	17.4 ± 3.0**	19.0 ± 3.3**	29.7 ± 15.7*	29.9 ± 13.0	51.8 ± 12.7	<10^–9^	
L-BSE	Mo	PBS	19.9 ± 2.8	31.2 ± 4.7	42.7 ± 7.7	58.6 ± 2.9	58.4 ± 4.7	>60.0	10^–8^	<10^–4^
		NBH	>60.0**	>60.0**	>60.0**	>60.0	>60.0	>60.0	>10^–4^	
	Bv	PBS	12.3 ± 0.7	23.0 ± 7.6	28.7 ± 9.5	37.6 ± 9.7	48.6 ± 10.6	56.6 ± 4.5	<10^–9^	<10^–5^
		NBH	>60.0**	>60.0**	>60.0**	>60.0**	>60.0**	> 60.0*	>10^–4^	
	Ha	PBS	21.4 ± 2.9	47.7 ± 9.0	44.8 ± 12.8	55.1 ± 8.6	>60.0	>60.0	10^–7^	<10^–3^
		NBH	>60.0**	>60.0**	>60.0**	>60.0	>60.0	>60.0	>10^–4^	
	Bo	PBS	51.3 ± 10.4	52.4 ± 5.9	58.8 ± 3.7	>60.0	>60.0	>60.0	10^–6^	<10^–2^
		NBH	>60.0*	>60.0**	>60.0	>60.0	>60.0	>60.0	>10^–4^	
	Sh	PBS	19.3 ± 4.1	30.7 ± 6.9	32.8 ± 9.9	52.0 ± 9.0	57.7 ± 4.9	> 60.0	10^–8^	10^–1^
		NBH	31.1 ± 12.3*	47.2 ± 15.6*	50.9 ± 18.0*	59.1 ± 2.8*	> 60.0	> 60.0	10^–7^	
	Cer	PBS	13.7 ± 2.1	19.5 ± 4.0	25.1 ± 6.1	36.8 ± 11.9	57.5 ± 5.9	57.5 ± 7.4	<10^–9^	1
		NBH	18.6 ± 4.3**	22.5 ± 3.7	25.2 ± 7.2	36.2 ± 12.8	56.9 ± 6.2	58.6 ± 4.1	<10^–9^	
C-BSE	Mo	PBS	26.2 ± 10.9	33.7 ± 14.8	50.0 ± 13.0	>60.0	>60.0	59.8 ± 0.5	10^–6^	<10^–2^
		NBH	>60.0**	>60.0**	>60.0*	>60.0	>60.0	>60.0	>10^–4^	
	Bv	PBS	23.8 ± 7.5	54.7 ± 8.6	59.9 ± 0.3	>60.0	>60.0	>60.0	10^–6^	<10^–2^
		NBH	>60.0**	>60.0	>60.0	>60.0	>60.0	>60.0	>10^–4^	
	Ha	PBS	52.1 ± 13.2	53.4 ± 9.9	>60.0	>60.0	>60.0	>60.0	10^–5^	<10^–1^
		NBH	>60.0	>60.0	>60.0	>60.0	>60.0	>60.0	>10^–4^	
	Bo	PBS	53.6 ± 6.5	>60.0	>60.0	>60.0	>60.0	>60.0	10^–4^	<1
		NBH	>60.0**	>60.0	>60.0	>60.0	>60.0	>60.0	>10^–4^	
	Sh	PBS	34.5 ± 3.9	49.1 ± 9.5	59.6 ± 1.2	>60.0	>60.0	>60.0	10^–6^	<10^–2^
		NBH	>60.0**	>60.0**	>60.0	>60.0	>60.0	>60.0	>10^–4^	
	Cer	PBS	20.7 ± 2.5	38.7 ± 7.5	56.5 ± 8.0	>60.0	>60.0	>60.0	10^–6^	10^–2^
		NBH	53.5 ± 12.9**	>60.0**	>60.0	>60.0	>60.0	>60.0	10^–4^	

^a^Seeds (brain homogenates from prion-infected animal) were serially diluted 10-fold with either PBS or 2% normal brain homogenate (NBH) (final concentration in the reaction mixture was 0.1%) of the same species as the seeds.

^b^Mean ± SD from three independent experiments with three replicates are shown (*: p < 0.05, **: p < 0.01, welch t-test).

^c^The ratio was calculated as end-point^PBS^/end-point^NBH^.

### Involvement of N-terminal region of rCerPrP in its reactivity in the presence of NBH

**Figure 2.**
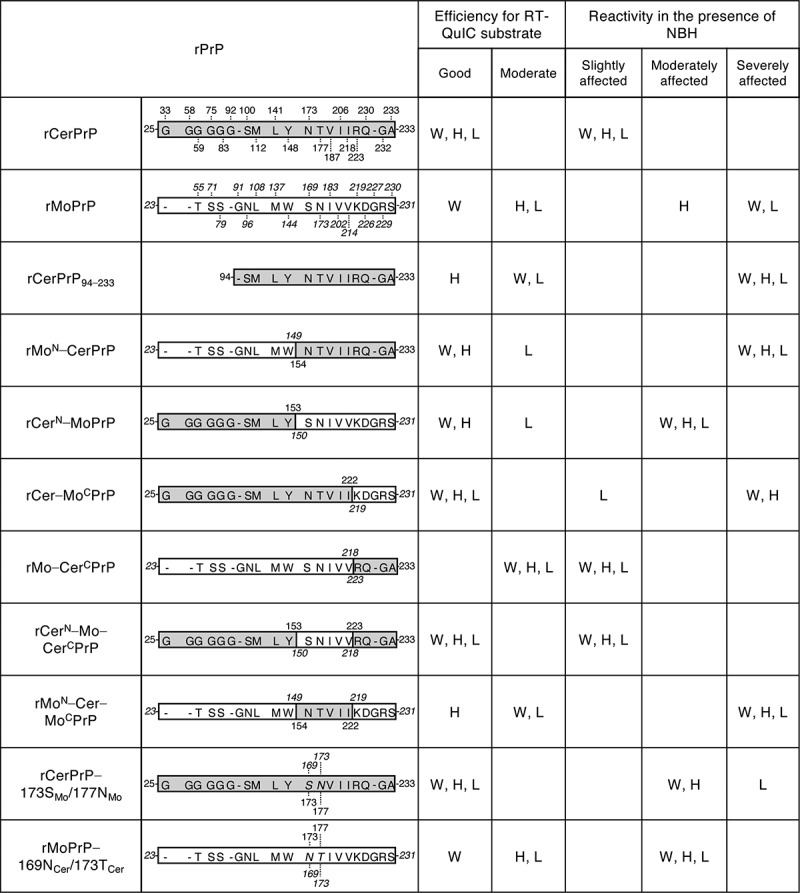
Summary of the reactivity of recombinant chimeric PrPs between CerPrP and MoPrP. rCerPrP, rMoPrP, and their chimeras are indicated with their amino acid differences. Numbers with capital letters are the aa of CerPrP, while those with italics indicate the aa of MoPrP. ‘-’ indicates gaps. A single-letter notation of aa with italics indicate the substituted aa between CerPrP and MoPrP. The RT-QuIC substrate efficiencies were classified as follows: Good, detection endpoints are ≦10^−8^ and lag phases at 10^−4^ and 10^−5^ seed dilutions were <20 h for seeds diluted with PBS; Moderate, detection endpoints were >10^−8^ or lag phases at 10^−4^ or 10^−5^ seed dilutions were ≧20 h. The criteria of the reactivity in the presence of NBH were defined as follows: slightly affected, endpoint ratio was ≧10^–2^ and prolongation of the lag phase at 10^−5^ seed dilution (lag phase^NBH^ − lag phase^PBS^) was <10 h; moderately affected: endpoint ratio is 10^−2^ to 10^−3^ and/or prolongation of the lag phase at 10^−5^ was from 10–25 h; severely affected, endpoint ratio was <10^−3^ and/or prolongation of the lag phase at 10^−5^ seed dilution is >25 h. H, H-BSE; L, L-BSE; W, CWD

RT-QuIC reaction is reported to be easily inhibited by high concentrations of tissue homogenates and body fluids [31,33]. In the current study, we found that the reaction of rCerPrP to CWD and atypical BSE prions was not largely affected by NBH. To clarify the mechanism of the unique property of rCerPrP, we analysed the reactivities of rPrPs shown in Figure 2. The reactions of N-terminal-truncated rCerPrP comprised aa 94–233 (rCerPrP_94–233_) to CWD, and atypical BSE prions were completely inhibited by NBH (Table 2). Furthermore, lag phases for detecting CWD and L-BSE prions using rCerPrP_94–233_ at the 10^−4^ seed dilution in PBS containing 0.01% brain homogenates were obviously longer than those using full-length rCerPrP. Replacement of the N-terminal region of rCerPrP (aa 25–153) with the corresponding region of rMoPrP (aa 23–149) (rMo^N^–CerPrP) had modest influence on the detection endpoints for CWD and atypical BSE prions: the detection endpoints of rMo^N^–CerPrP for CWD, H-BSE, and L-BSE (10^−8^, 10^−8^, and 10^−7^, respectively, without NBH, Table 3) were slightly lower than those of rCerPrP (10^−8^, <10^−9^, and <10^−9^, respectively, Figure 1 and Table 1). Additionally, the reaction of rMo^N^–CerPrP to the L-BSE prions was completely inhibited, and reactions of rMo^N^–CerPrP to CWD and H-BSE were severely affected by 0.1% NBH; the detection endpoints for CWD, H-BSE, and L-BSE prions worsened by 4, 2, and >3 logs, respectively, with significant prolongation of lag phases at each seed dilution (Table 3). The reactivities of rMo^N^–CerPrP were very similar to those of rMoPrP; the reaction of rMoPrP to the L-BSE prions were completely inhibited (Table 1), and the reactions of rMoPrP to CWD (Figure 1) and H-BSE prions (Table 1) were severely affected as observed in the significant prolongation of lag phases in the presence of 0.1% NBH.

**Table 2.** Reactivity of N-terminal truncated rCerPrP in RT-QuIC

rPrP	Seed	Diluent^(^^a)^	Lag phase (h)^(^^b)^						End-point	Ratio^(^^c)^
			10^–4^	10^–5^	10^–6^	10^–7^	10^–8^	10^–9^		
rCerPrP _94–233_	CWD	PBS	38.1 ± 16.7	21.0 ± 10.4	23.6 ± 13.1	30.7 ± 19.9	48.1 ± 15.1	56.2 ± 11.3	<10^–9^	<10^–5^
		NBH	>60.0**	>60.0**	>60.0**	>60.0**	>60.0*	>60.0	>10^–4^	
	H-BSE	PBS	14.1 ± 7.3	10.8 ± 5.5	11.6 ± 1.2	14.1 ± 7.2	29.7 ± 19.8	>60.0	10^–8^	<10^–4^
		NBH	>60.0**	>60.0**	>60.0**	>60.0**	>60.0**	>60.0	>10^–4^	
	L-BSE	PBS	23.8 ± 17.1	18.3 ± 3.2	29.2 ± 11.9	47.3 ± 15.6	57.9 ± 4.8	>60.0	10^–8^	<10^–4^
		NBH	>60.0**	>60.0**	>60.0**	>60.0*	>60.0	>60.0	>10^–4^	

^a^^–c^ Descriptions are the same as Table 1.

**Table 3.** Reactivity of recombinant chimeric PrP between CerPrP and MoPrP in RT-QuIC

rPrP	Seed	Diluent^(^^a)^	Lag phase (h) ^(^^b)^						End-point	Ratio ^(^^c)^
			10^–4^	10^–5^	10^–6^	10^–7^	10^–8^	10^–9^		
rMo^N^– CerPrP	CWD	PBS	10.8 ± 1.9	14.3 ± 2.2	19.2 ± 5.5	23.6 ± 9.6	53.2 ± 13.7	>60.0	10^–8^	10^–4^
		NBH	46.8 ± 9.1**	>60.0**	>60.0**	>60.0**	>60.0	>60.0	10^–4^	
	H-BSE	PBS	7.8 ± 1.4	10.5 ± 2.2	12.6 ± 2.3	17.4 ± 2.8	34.6 ± 21.0	>60.0	10^–8^	10^–2^
		NBH	52.4 ± 13.5**	58.1 ± 4.5**	58.8 ± 3.6**	>60.0**	>60.0**	>60.0	10^–6^	
	L-BSE	PBS	13.5 ± 2.7	18.2 ± 2.2	28.1 ± 5.6	44.9 ± 14.0	>60.0	>60.0	10^–7^	<10^–3^
		NBH	>60.0**	>60.0**	>60.0**	>60.0**	>60.0	>60.0	>10^–4^	
rCer^N^–MoPrP	CWD	PBS	8.1 ± 1.2	10.0 ± 1.5	11.8 ± 2.1	17.4 ± 6.0	45.1 ± 17.3	>60.0	10^–8^	1
		NBH	20.6 ± 2.0**	21.8 ± 3.0**	23.9 ± 2.5**	34.4 ± 8.6**	51.5 ± 12.0	>60.0	10^–8^	
	H-BSE	PBS	14.2 ± 2.1	17.6 ± 1.4	19.8 ± 2.0	25.0 ± 3.3	29.0 ± 3.3	51.1 ± 10.4	<10^–9^	1
		NBH	28.4 ± 9.2**	38.5 ± 12.9**	35.7 ± 12.5**	44.7 ± 9.2**	54.2 ± 11.2**	57.1 ± 8.8	<10^–9^	
	L-BSE	PBS	13.8 ± 2.1	20.1 ± 3.8	28.8 ± 11.3	45.1 ± 12.8	58.2 ± 5.5	>60.0	10^–8^	1
		NBH	35.3 ± 16.9**	32.1 ± 15.3*	44.0 ± 18.9	47.1 ± 13.0	58.2 ± 3.9	>60.0	10^–8^	
rCer – Mo^C^PrP	CWD	PBS	10.6 ± 1.6	12.5 ± 2.0	13.6 ± 2.7	19.6 ± 3.6	33.6 ± 12.6	58.0 ± 4.5	<10^–9^	<10^–3^
		NBH	41.0 ± 15.9**	44.6 ± 14.5**	57.9 ± 6.2**	>60.0**	>60.0**	>60.0	10^–6^	
	H-BSE	PBS	9.5 ± 1.3	10.6 ± 2.8	13.0 ± 2.5	26.7 ± 12.8	33.3 ± 15.9	54.7 ± 10.7	<10^–9^	<10^–1^
		NBH	43.8 ± 15.2**	54.5 ± 10.6**	57.8 ± 6.7**	58.5 ± 4.6**	57.0 ± 9.1**	>60.0	10^–8^	
	L-BSE	PBS	12.6 ± 1.8	19.7 ± 1.8	24.0 ± 2.5	41.5 ± 8.4	57.6 ± 2.4	58.9 ± 2.4	<10^–9^	1
		NBH	28.8 ± 4.9**	27.6 ± 6.5**	36.7 ± 11.8*	52.5 ± 9.6*	57.8 ± 6.6	57.8 ± 6.7	<10^–9^	
rMo – Cer^C^PrP	CWD	PBS	20.5 ± 3.5	24.5 ± 7.1	29.7 ± 6.7	33.4 ± 7.5	52.2 ± 11.6	58.9 ± 2.5	<10^–9^	1
		NBH	20.4 ± 2.2	23.7 ± 2.9	30.3 ± 12.4	33.6 ± 5.6	45.5 ± 9.5	59.6 ± 1.2	<10^–9^	
	H-BSE	PBS	22.9 ± 10.9	26.5 ± 6.2	29.7 ± 11.0	28.9 ± 6.4	51.6 ± 9.6	58.5 ± 3.0	<10^–9^	1
		NBH	22.3 ± 1.4	35.8 ± 12.8	37.6 ± 12.8	41.7 ± 13.3*	48.5 ± 8.5	59.1 ± 2.7	<10^–9^	
	L-BSE	PBS	22.0 ± 5.0	25.7 ± 6.8	35.9 ± 9.1	44.6 ± 9.1	51.6 ± 18.4	>60.0	10^–8^	1
		NBH	25.4 ± 6.7	33.3 ± 6.3*	43.8 ± 8.1	50.1 ± 7.9	59.3 ± 1.5	>60.0	10^–8^	
rCer^N^ – Mo–Cer^C^PrP	CWD	PBS	12.5 ± 2.6	14.7 ± 2.0	20.1 ± 5.2	28.9 ± 12.4	52.6 ± 15.6	>60.0	10^–8^	1
		NBH	17.2 ± 2.3**	20.3 ± 3.5**	22.9 ± 4.3	34.5 ± 4.8	53.7 ± 6.8	>60.0	10^–8^	
	H-BSE	PBS	13.3 ± 0.8	16.6 ± 2.0	17.4 ± 1.1	22.6 ± 3.8	38.8 ± 12.0	56.5 ± 9.4	<10^–9^	1
		NBH	17.2 ± 2.5**	22.1 ± 3.9**	27.0 ± 3.3**	32.8 ± 4.5**	42.0 ± 6.6	52.9 ± 7.9	<10^–9^	
	L-BSE	PBS	11.7 ± 0.9	19.5 ± 3.5	23.6 ± 2.9	37.9 ± 12.8	55.7 ± 6.9	59.8 ± 0.7	<10^–9^	<10^–1^
		NBH	17.3 ± 3.2**	25.4 ± 5.0**	33.7 ± 6.5**	41.4 ± 14.0	59.5 ± 1.6	>60.0	10^–8^	
**rPrP**	**Seed**	**Diluent^(^**^**a)**^	**Lag phase (h)** **^(^**^**b)**^						**End-point**	**Ratio ^(^^c)^**
			**10^−4^**	**10^−5^**	**10^−6^**	**10^−7^**	**10^−8^**	**10^−9^**		
rMo^N^ – Cer–Mo^C^ PrP	CWD	PBS	13.8 ± 2.4	21.8 ± 5.4	22.7 ± 3.0	29.6 ± 8.6	57.3 ± 5.7	59.6 ± 1.2	<10^–9^	<10^–5^
		NBH	>60.0**	>60.0**	>60.0**	>60.0**	>60.0	>60.0	>10^–4^	
	H-BSE	PBS	9.4 ± 1.6	14.6 ± 5.1	19.5 ± 7.0	31.2 ± 13.5	47.0 ± 15.3	>60.0	10^–8^	<10^–4^
		NBH	>60.0**	>60.0**	>60.0**	>60.0**	>60.0*	>60.0	>10^–4^	
	L-BSE	PBS	17.6 ± 4.1	22.3 ± 3.0	29.2 ± 9.6	46.9 ± 11.4	55.7 ± 5.7	56.4 ± 6.1	<10^–9^	<10^–5^
		NBH	>60.0**	>60.0**	>60.0**	>60.0**	>60.0*	>60.0	>10^–4^	
rCerPrP–173S_Mo_/177N_Mo_	CWD	PBS	9.5 ± 0.7	11.3 ± 1.0	14.4 ± 3.1	26.3 ± 12.8	49.6 ± 14.6	57.2 ± 5.9	<10^–9^	<10^–2^
		NBH	32.8 ± 8.2**	36.1 ± 13.9**	47.5 ± 10.8**	55.5 ± 8.0*	>60.0*	>60.0	10^–7^	
	H-BSE	PBS	9.5 ± 1.0	11.3 ± 0.9	13.5 ± 1.2	18.8 ± 3.1	27.4 ± 14.4	48.1 ± 11.1	<10^–9^	<10^–2^
		NBH	30.7 ± 11.3**	42.8 ± 15.2**	54.5 ± 12.9**	54.4 ± 9.8**	>60.0**	>60.0**	10^–7^	
	L-BSE	PBS	12.1 ± 3.1	17.6 ± 7.6	29.9 ± 17.8	32.4 ± 12.2	>60.0	56.5 ± 5.1	10^–9^	10^–1^
		NBH	31.1 ± 9.3**	48.1 ± 13.7**	49.6 ± 14.0*	>60.0**	56.8 ± 9.7	>60.0	10^–8^	
rMoPrP–169N_Cer_/ 173T_Cer_	CWD	PBS	12.6 ± 2.4	13.2 ± 1.7	18.5 ± 3.9	34.8 ± 19.5	53.1 ± 15.9	59.5 ± 1.4	<10^–9^	<10^–1^
		NBH	28.2 ± 4.9**	37.4 ± 7.9**	49.3 ± 12.0**	58.4 ± 4.3**	59.1 ± 2.7	>60.0	10^–8^	
	H-BSE	PBS	11.9 ± 3.8	27.0 ± 14.9	31.9 ± 5.2	45.1 ± 14.9	55.3 ± 9.3	>60.0	10^−–8^	1
		NBH	20.1 ± 8.8*	42.6 ± 16.4	53.5 ± 10.5**	56.8 ± 9.6	55.7 ± 13.0	>60.0	10^–8^	
	L-BSE	PBS	23.5 ± 5.2	24.9 ± 9.3	32.8 ± 13.2	46.9 ± 16.6	59.7 ± 0.9	>60.0	10^–8^	10^–1^
		NBH	21.6 ± 7.2	49.4 ± 10.3**	59.7 ± 1.0**	59.5 ± 1.6	> 60.0	>60.0	10^–7^	

^a–c^Descriptions are the same as Table 1.

On the contrary, replacing the N-terminal half of MoPrP (aa 23–149) with the corresponding CerPrP (aa 25–153) (rCer^N^–MoPrP) partially restored reactivity to a level similar to that of rCerPrP. Detection endpoints of CWD, H-, and L-BSE prions using rCer^N^–MoPrP without NBH (10^−8^, <10^−9^, and 10^−8^, respectively, Table 3) were almost comparable to those using rCerPrP (10^−8^, <10^−9^, and <10^−9^, respectively, Figure 1 and Table 1). In addition, the reactivity of rCer^N^–MoPrP to CWD, H-, and L-BSE prions was not affected by NBH when endpoints were compared in the absence (PBS) and presence of NBH (Table 3). However, the prolongation of lag phases in the detection of CWD, H-, and L-BSE prions using rCer^N^–MoPrP (11.8, 20.9, and 12.0 h, respectively, at 10^−5^ seed dilution, Table 3) appeared to be longer than those caused by rCerPrP in the presence of NBH (7.7, 6.5, and 3.0 h, respectively, at 10^−5^ seed dilution, Figure 1 and Table 1), suggesting that rCer^N^–MoPrP is more affected by NBH than by rCerPrP. Taken together, these results suggest that the N-terminal region of rCerPrP is necessary for its unique reactivity in RT-QuIC in the presence of NBH.

### Involvement of C-terminal region of rCerPrP in its reactivity in the presence of NBH

It was reported that the aa sequence at the C-terminus of PrP^C^ is varied among animal species [34], and aa substitution in this region could alter the efficacy of PrP^C^ conformational conversion [35,36] and transmission kinetics [37]. Thus, we analysed the involvement of the C-terminal region of CerPrP in its reactivity in RT-QuIC. The rCer–Mo^C^PrP, which possesses MoPrP aa 219–231 in the corresponding region of rCerPrP aa 223–233 resulting in 5 amino acid differences, reacted well with CWD, H-, and L-BSE prions (endpoints: <10^−9^, Table 3) in the absence of NBH if compared with the reactivity of rCerPrP (10^−8^, <10^−9^, and <10^−9^, respectively, Figure 1 and Table 1). Lag phases for the detection of the three prions using rCer–Mo^C^PrP (12.5, 10.6, and 19.7 h, respectively, at 10^−5^ seed dilution, Table 3) were comparable to those using rCerPrP (10.0, 10.9, and 19.5 h, respectively, Figure 1 and Table 1). However, the detection endpoint of CWD prions using rCer–Mo^C^PrP worsened by >3 log in the presence of 0.1% NBH with prolonged lag phases (Table 3). This reactivity is similar to that of rMoPrP to CWD prions (Figure 1). Although the detection endpoint of H-BSE prions using rCer–Mo^C^PrP worsened only by 1 log in the presence of 0.1% NBH, lag phases for the detection of H-BSE prions using rCer–Mo^C^PrP were significantly prolonged, e.g, lag phases for detecting H-BSE prions using rCerPrP were prolonged by only 6.5 h at 10^−5^ seed dilution (Table 1), but those using rCer–Mo^C^PrP were prolonged by 43.9 h at 10^−5^ seed dilution in the presence of 0.1% NBH (Table 3). On the contrary, rCer–Mo^C^PrP reactivity to L-BSE was not severely affected, and no differences in the detection endpoints with only marginal prolongation of lag phases (around 10 h) at each seed dilutions (Table 3). Thus, the reactivity of rCer–Mo^C^PrP to CWD and H-BSE prions in the presence of NBH resembles that of rMoPrP, whereas, its reactivity to L-BSE prions resembles that of rCerPrP. Replacement of MoPrP aa 219–231 with the corresponding CerPrP 223–233 (rMo–Cer^C^PrP) did not affect the detection endpoints of CWD, H-, and L-BSE prions diluted with PBS; <10^−9^, <10^−9^, and 10^−8^, respectively (Table 3). However, lag phases in the detection of three prions diluted with PBS using rMo–Cer^C^PrP (about 20.5–26.5 h at 10^−4^ and 10^−5^ seed dilutions, Table 3) were longer than those using rCerPrP (less than 19.5 h at 10^−4^ and 10^−5^ seed dilutions, Figure 1 and Table 1), but were comparable to those using rMoPrP (14.7–31.2 h at 10^−4^ and 10^−5^ seed dilutions, Figure 1 and Table 1), suggesting that rMo–Cer^C^PrP is less efficient than rCerPrP as a substrate. Interestingly, only eleven amino acids in the C-terminal region of CerPrP appeared to confer the unique property of rCerPrP to rMoPrP that made it such that the presence of NBH interferes less with RT-QuIC using rCerPrP. The reactivity of rMoPrP to the three prions was severely interfered in the presence of 0.1% NBH. Detection endpoints worsened by 2–4 logs with a significant prolongation of the lag phases (43.5, 27.0, and >28.8 h for CWD, H-, and L-BSE, respectively, at 10^−5^ seed dilutions, Figure 1 and Table 1). However, those of rMo–Cer^C^PrP were less affected by 0.1% NBH; their detection endpoints were not changed, but with a slight prolongation of the lag phases was observed (0, 9.3, and 7.6 h, respectively), at 10^−5^ seed dilutions (Table 3). rCer^N^–Mo–Cer^C^PrP, which is a chimeric PrP composed of CerPrP aa 25–153, MoPrP aa 150–218, and CerPrP aa 223–233, was detected well with CWD, H-, and L-BSE prions without worsening the detection endpoints but had a marginal prolongation of the lag phases, with most of them being less than 10 h long in the presence of NBH (Table 3). These results were consistent with the finding that both N- and C-terminal regions of CerPrP are involved in its unique reactivity in RT-QuIC in the presence of NBH. On the contrary, rMo^N^–Cer–Mo^C^PrP, which has an opposite structure to rCer^N^–Mo–Cer^C^PrP, showed similar reactivity to rMoPrP, and detection of CWD, H-, and L-BSE prions was severely affected by NBH (Table 3).

### Effect of CerPrP-specific amino acids in the β2–α2 loop on reactivity in the presence of NBH

Several studies reported that the CerPrP-specific aa Asn173 and Thr177 in the β2–α2 loop (aa 168–178) [38] influence cross-species prion transmission in transgenic mice and affect the reaction of PMCA [39,40]. To clarify the influence of these aa residues on the unique reactivity of rCerPrP in RT-QuIC, we analysed the reactivities of rCerPrP carrying the corresponding MoPrP aa Ser169 and Ans173 (rCerPrP–173S_Mo_/177N_Mo_). Substitution of two CerPrP-specific aa did not affect the detection endpoints of the CWD and atypical BSE prions (10^−9^ or <10^−9^, in Table 3), compared with rCerPrP (10^−8^, <10^−9^, and <10^−9^, for CWD, H-, and L-BSE prions, respectively, Figure 1 and Table 1) in the absence of NBH. However, substitutions of the two amino acids affected the reactivity of rCerPrP in the presence of NBH: the detection endpoints worsened by more than >2 log for the CWD and H-BSE prions and by 1 log for L-BSE prions, with >20 h-prolonged lag phases at most seed dilutions (Table 3). The substitution of MoPrP Ser169 and Asn173 to the corresponding CerPrP Asn173 and Thr177 (rMoPrP–169N_Cer_/173T_Cer_) reduced the reactivity to H- and L-BSE prions by >1 log in the absence of NBH. The reactivity of rMoPrP to CWD, H-, and L-BSE prions were extremely affected in the presence of NBH as described above; however, detection of three prions using rMoPrP–169N_Cer_/173T_Cer_ in the presence of NBH was moderately affected compared to rMoPrP, with the detection endpoints reduced by only 1 log for CWD and L-BSE prions with moderate prolongation of lag phases (8.2–24.5 h at 10^−4^ and 10^−5^ seed dilutions, Table 3).

## Discussion

**Figure 3.**
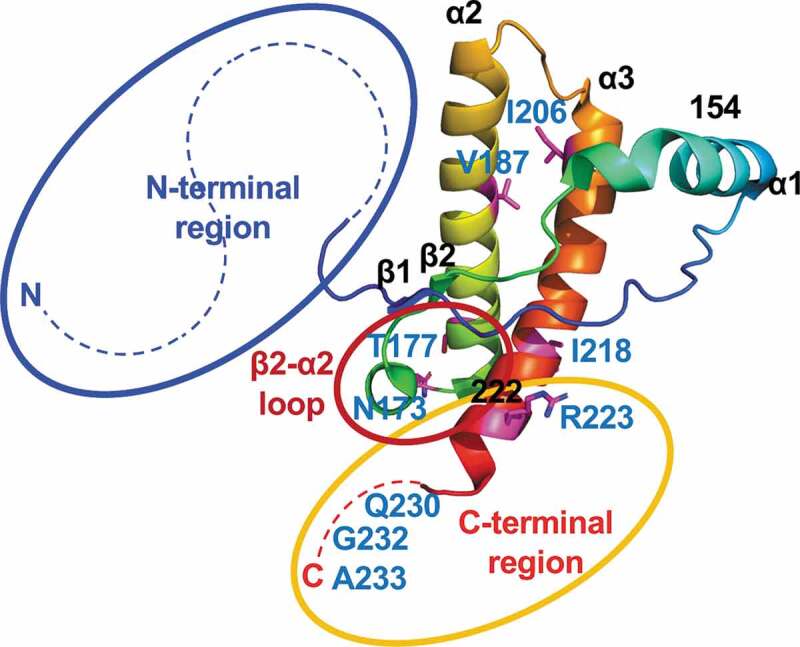
Regions responsible for the unique reactivity of rCerPrP in RT-QuIC. The three-dimensional structure of CerPrP (PDB ID: 4YXH) was drawn using open-source PyMOL. The regions responsible for the unique reactivity of rCerPrP, N- and C-terminal regions and the β2–α2 loop are shown using blue, yellow, and red circles, respectively. The aa of rCerPrP in the β2–α2 loop and C-terminus are shown in the single-letter notation. Hydrophobic aa differed between rCer^N^–Mo–Cer^C^PrP and rCerPrP–173S_Mo_/177N_Mo_ and regions in which rCerPrP are replaced with (aa 153–222) are shown around the structure. The side chains of aa with aa numbers except for three aa at C-terminal end were drawn with sticks and the aa residues were coloured indicated with magenta

rPrPs from Bv, Ha, and human (Hu) have been widely used as substrates to detect the seeding activity of various prions by the RT-QuIC reaction [28–31]. However, few reports are available regarding the utility of CerPrP. The reaction is known to be sensitive to inhibitory factor(s) present in tissue homogenates and body fluids [31–33]. Concentration of PrP^Sc^ through immunoprecipitation or iron oxide beads, or lipid removal of through alcohol extraction, have been attempted to reduce the influence of inhibitory factors [32,41,42]; however, a simpler method is still desirable for practical use. In the present study, we demonstrated that rCerPrP reacted with CWD and atypical BSE prions, even in the presence of the highest concentration of NBH (0.1%), in the reaction mixture. Since rCerPrP reactivity was stable and reproducible without the influence of lot differences, we attempted to determine the region(s) responsible for the unique reactivity of rCerPrP. The unique reactivity of rCerPrP disappeared when the N-terminal region (aa 25–93) was truncated. Moreover, replacement of the N-terminal half of MoPrP with the corresponding region of CerPrP partially restored this reactivity, but replacing the N-terminal half of CerPrP with the corresponding region of MoPrP abolished the unique reactivity of rCerPrP (summarized in Figure 2). These results indicate that the N-terminal region of the rCerPrP is one of the determinants modulating rCerPrP reactivity in the RT-QuIC reaction in the presence of NBH (Figure 3). N-terminal truncated rPrPs have been known to act as better substrates for detecting the amyloid seeding activity of PrP^Sc^ [30,43]. However, it has been reported that the N-terminal region is one of the essential regions for the binding between PrP^C^ and PrP^Sc^, and PrP^Sc^ production [35,44–46]. Deletion of the octapeptide repeat region (aa 51–90 of MoPrP) delayed the accumulation of PrP^Sc^ and prolonged the survival of mice inoculated with C-BSE prions [45], suggesting the involvement of the N-terminal region in the efficacy of conformational conversion in certain combinations of PrP^C^ and PrP^Sc^. Amino acid polymorphisms of CerPrP at Gln95 and Gly96 are known to modulate the susceptibility of deer to CWD prions [47,48], also suggesting the influence of the N-terminal region of cervid PrP^C^ on the efficacy of its conformational conversion. There are two possibilities regarding the role of the CerPrP N-terminal region in its unique reactivity in the presence of NBH: first, the presence of the N-terminal region may stabilize intra- or inter-molecular interaction, which enhances the efficacy of amyloid formation. Alternatively, the N-terminal region effectively inhibits the interaction of inhibitory factor(s) in tissue homogenates with rCerPrP; however, the latter is unlikely because the reactivity of rCerPrP to C-BSE was affected in the presence of NBH.

Although rMo–Cer^C^PrP was not a good substrate, i.e., the lag phases for detecting CWD and atypical BSE prions were >20 h at 10^−4^ and 10^−5^ seed dilutions (Table 3), the C-terminal region of rCerPrP is also involved in the unique reactivity of rCerPrP, and the reactivity of rMo–Cer^C^PrP in RT-QuIC was less affected with NBH than that of rMoPrP (Figure 2 and Table 3). However, the unique reactivity of rCerPrP in the presence of NBH disappeared due to N-terminal truncation even in the presence of the C-terminal region of CerPrP (rCerPrP_94–233_) (Figure 2 and Table 2), suggesting that a cooperative effect of the C-terminal region with the N-terminal region. Indeed, rCer^N^–Mo–Cer^C^PrP showed better reactivity than rCer–Mo^C^PrP when used as a substrate and was less affected in the presence of NBH compared to rCer–Mo^C^PrP, suggesting an additive effect of the N- and C-terminal regions of CerPrP (Figure 2). The NMR structure of rHuPrP and rMoPrP revealed the intramolecular interaction between the C-terminal region (aa 219–226) and the N-terminal flexible region [49] or the C-terminal region (aa 215–223) and the β2–α2 loop (aa 164–174) [34]. These intramolecular interactions could be destabilized by substituting Gln217_Mo_ to Arg, which is a mutation associated with GSS [50], and HuPrP Glu219 to Lys, which is a protective polymorphism against sporadic CJD [51]. Thus, intra- or inter-molecular interactions between the N- and C-terminal regions of rCerPrP may be strong enough to overcome the influence of possible inhibitor(s) in NBH and to promote the conformational conversion of rCerPrP.

Since rCer^N^–Mo–Cer^C^PrP possessed five amino acids from MoPrP including amino acid substitutions at aa 173 and aa 177, we expected that the reactivity to CWD and to atypical BSE prions of rCerPrP–173S_Mo_/177N_Mo_, which possesses only two amino acid differences from CerPrP, would be closer to that of rCerPrP compared to that of rCer^N^–Mo–Cer^C^PrP. The detection endpoints of rCerPrP–173S_Mo_/177N_Mo_ for detecting CWD and atypical BSE prions were comparable to that of rCer^N^–Mo–Cer^C^PrP when the seeds were diluted with PBS. However, the reactivity of rCerPrP–173S_Mo_/177N_Mo_ was more susceptible to the inhibitory effect of NBH than to that of rCer^N^–Mo–Cer^C^PrP even though the N- and C-terminal regions of rCerPrP–173S_Mo_/177N_Mo_ were composed of CerPrP (Figure 2). One possible explanation for this may be an incompatibility of amino acids in the aa 154–222 region of CerPrP, which contains five aa differences between Cer and MoPrP. Indeed, aa 173 and 177 of rCerPrP–173S_Mo_/177N_Mo_ were substituted by Ser169 and Asn173 of MoPrP, respectively, which are located within the β2–α2 loop (aa 168–178 of CerPrP) [38], whereas the remaining three aa are Val187 (α2-helix: aa 176–197 of CerPrP), Ile206, and Ile218 (α3-helix: aa 202–226 of CerPrP) (Figure 3). An amino acid substitution at Ser170 of HuPrP with the corresponding Asn from BvPrP, which in turn corresponds to aa 173 of CerPrP, increased the conversion of HuPrP^C^ into the protease-resistant PrP (PrP-res) by CWD prions during protein PMCA analysis [36]. Rabbit (Rb) PrP is known to be difficult to convert into PrP-res; however, substitution at Ile202 of RbPrP using the corresponding Val from MoPrP, which corresponds to aa 206 of CerPrP, increased the conversion of RbPrP^C^ into PrP-res by RML prions during PMCA [35]. Additionally, substitution of Ile215 of HuPrP with the Val from BvPrP, which corresponds to aa 218 of CerPrP, worsened the conversion efficiency of HuPrP^C^ by the RML prions [36]. The β2–α2 loop is known to interact with the α3-helix through hydrophobic interactions and a disulphide bridge, but single amino acid substitutions within these regions decreased hydrophobic interactions [52]. Therefore, heterologous amino acid combinations in the β2–α2 loop and α3-helix may influence PrP stability and affect the conversion efficiency of rPrP during RT-QuIC.

In the current study, we showed that full-length rCerPrP as a substrate is useful for detecting CWD and atypical BSE prions in tissues with low level of prions, since the reaction of rCerPrP was not highly affected by high NBH concentrations. Additionally, we found that at least the N- and C-terminal regions of CerPrP are involved in the unique reactivity of the rCerPrP. These results will be useful for optimizing artificial rPrP for RT-QuIC reactions.

## Materials and methods

### Brain materials

Brain tissue from six CWD-affected deer was pooled and used as a source of CWD prions. Two brains of unaffected white-tail deer in USA were pooled and used as negative control [25]. Each tissue was homogenized in phosphate-buffered saline (PBS) at a concentration of 20% and was frozen at – 80°C until use. Brain homogenate of H-BSE-affected cattle [53] was kindly provided by Dr Iwamaru Y, National Institute of Animal Health, Japan. Ten-percent of brain homogenates from C-BSE [54] and L-BSE [55]-affected cattle were prepared with PBS and stored at – 80°C.

### Construction of the expression system of recombinant PrPs

Expression plasmids for the full-length recombinant mouse and hamster PrPs (rMoPrP, rHamPrP; amino acids (aa) 23–231) were kindly provided by Dr Atarashi R, Miyazaki University, Japan. An expression plasmid for the full-length bank vole rPrP (rBvPrP; aa 23–230) was kindly provided by Dr Caughey B, National Institute of Health, USA. Genes encoding for full-length bovine PrP (BoPrP; aa 25–242), CerPrP (aa 25–233, genotype: G_96_M_132_S_225_Q_226_ [56]) and sheep (ARQ) PrP (ShPrP) (aa 25–233) were amplified from the corresponding genome DNA (Supplementary Table 1). The gene fragment encoding N-terminal truncated rCerPrP (aa 94–233) was amplified from that of the full-length rCerPrP using primers listed in Supplementary Table 2. Each gene fragment was subcloned using the Zero Blunt TOPO PCR Cloning Kit (Invitrogen, USA). Nucleotide sequences were determined using BigDye v3.1 (Applied Biosystems, USA) and ABI-3130 Avant sequencer (Applied Biosystems). Each gene fragment with the correct nucleotide sequence was inserted into the NdeI and BamHI sites of pET11a (Novagen, USA).

Genes encoding the N-terminal replaced chimera of rCerPrP and rMoPrP (rMo^N^–CerPrP, rCer^N^–MoPrP) were generated using assembly PCR [57] with primer sets described in the Supplementary Table 2. Genes encoding the C-terminal replaced chimeras (rCer–Mo^C^PrP, rMo–Cer^C^PrP) were generated through two consecutive PCRs with primers in Supplementary Table 2. Genes encoding rCerPrP and rMoPrP with two amino acid substitutions (rCerPrP–173S_Mo_/177N_Mo_, rMoPrP–169N_Cer_/173T_Cer_) were generated by assembly PCR using primer sets listed in Supplementary Table 2. Each PrP gene was inserted into pET11a for the expression and purification of rPrPs.

### Expression and purification of rPrPs

Expression and purification of rPrPs were performed as described elsewhere [58] with minor modifications. Briefly, BL21(DE3)pLysS cells (Invitrogen) transformed with the expression plasmid were pre-cultured in 5 mL L-Broth medium overnight at 37°C, and were further cultured in 200 mL MagicMedia E. coli expression medium (Invitrogen) for 30 h at 37°C. Bacterial cells were lysed using CelLytic B cell lysis reagent (Sigma, USA) with lysozyme (Sigma) and benzonase (Millipore, USA) for 30 min at room temperature (r.t.). Inclusion bodies were collected by centrifugation at 14,400 × g for 10 min at 4°C. The resulting pellets were washed three times using the 10% CelLytic B cell lysis reagent and stored at −80°C until use. Pellets were thawed and solubilized in denaturing buffer (100 mM sodium phosphate [pH 8.0], 10 mM tris(hydoroxymethyl)aminomethane [tris], 6 M guanidine hydrochloride [GdnHCl]) by rotating for 2 h at r.t. After centrifugation at 5,170 × g for 30 min at 4°C, the supernatant was mixed with Ni-NTA superflow resin (Qiagen, Germany), equilibrated with the denaturing buffer on a rotator for 1 h at r.t., and the Ni-NTA superflow resin was loaded onto the XK16 column (GE Healthcare, UK). The rPrP was refolded using a linear gradient of 6 to 0 M GdnHCl in refolding buffer (100 mM sodium phosphate [pH 8.0], 10 mM tris) using the ÄKTAexplore 10S system (GE Healthcare) and eluted by a linear gradient of 0 to 500 mM imidazole in 10 mM tris (pH 5.8). The fractions were pooled and dialysed against ultra-pure water. After filtration, rPrP concentration was determined by measuring absorbance at 280 nm.

### RT-QuIC reaction

RT-QuIC with rMoPrP, rBvPrP, rBoPrP, rShPrP and rCerPrP was performed using reaction conditions described elsewhere [22]. The reaction using rHaPrP was performed with 60 μg/ml substrate and 350 mM NaCl without SDS. Brain homogenates (20%) from uninfected deer or cattle (hereafter referred to as normal brain homogenate [NBH]) were diluted to 2% with PBS and were used as the negative control or diluent for the seed of the corresponding species. Then, 2% BHs of prion-infected animals were prepared from stock solutions (10% or 20%) (Supplementary Figure 1) and were serially diluted with PBS or species-matched 2% NBH, and 5 μl of each dilution was added into the three wells as seed. Final concentrations of brain tissue homogenates of the seed in the reaction mixture were from 10^−4^ [0.01%] to 10^−9^ [0.0000001%] after diluting with PBS. When the seeds were diluted with 2% NBH, the final concentration of the NBH in the reaction was 0.1%. Reactions were performed using the Infinite F200 microplate reader (TECAN, Switzerland) at 37°C, or at 42°C for rBvPrP, using the same cycles as described elsewhere [22].

### Data analysis

Thresholds of the reactions were calculated as mean thioflavin T (ThT) fluorescence intensity plus 5 × SD from the negative control wells without seed. The reactions were considered positive when the ThT fluorescence intensity exceeded the threshold [59], with the following exception: if ThT fluorescence intensity temporarily exceeded the threshold within the first 1 h, the data from the first 1 h were excluded from calculations. If the oscillated waveforms were continuously observed throughout the reaction, it was considered as negative even the intensity exceeded the threshold. If oscillated waveforms were observed prior to the appearance of the typical ThT fluorescence curve observed from rPrP fibrils, the reaction was judged as positive once the typical fluorescence curve exceeded the threshold. The endpoint of the reaction was determined as the highest seed dilution that gave a positive reaction in three independent experiments with three replicates. If positive reactions were observed at 10^−9^ dilution, the endpoint was set as <10^−9^. The lag phase (h) was defined as the time required for the fluorescence intensity to exceed the threshold.

## Supplementary Material

Supplemental Material
