# Supplementary material for: Involvement of N- and C-terminal region of recombinant cervid prion protein in its reactivity to CWD and atypical BSE prions in real-time quaking-induced conversion reaction in the presence of high concentrations of tissue homogenates
Source: Prion. 2020 Dec 19;14(1):283–95. doi: 10.1080/19336896.2020.1858694 (PMC7757825; doi:10.1080/19336896.2020.1858694)
Supplement: Supplemental Material [file KPRN_A_1858694_SM7093.docx]

**Supplementary Table 1.** Primers for cloning of the full-length rPrPs.

| Constructs | Primer names | Nucleotide sequences ^b)^ |
| --- | --- | --- |
| rBoPrP | CerPrP-F ^a)^ | 5’-TTCATATGAAGAAGCGACCAAAACCTG-3’ |
|  | BoPrP-R | 5’-TTGGATCCTCATGCCCCTCGTTGGTAATA-3’ |
| rCerPrP | CerPrP-F ^a)^ | 5’-TTCATATGAAGAAGCGACCAAAACCTG-3’ |
|  | CerPrP-R | 5’-TTGGATCCTCATGCCCCTCTTTGGTAA-3’ |
| rShPrP | CerPrP-F ^a)^ | 5’-TTCATATGAAGAAGCGACCAAAACCTG-3’ |
|  | ShPrP-R | 5’-TTGGATCCTCATGCCCCCCTTTGGTAA-3’ |

^a^ Same forward primer was used for the amplification because the nucleotide sequence encoding aa 25-30 of PrP from three artiodactyls is identical.

^b^ Underlines in forward primers indicate NdeI site, whereas those in reverse primers indicate BamHI site.

**Supplementary Table 2.** Primers for constructing Cer/Mo chimeric PrPs.

| Constructs | Primer names | Nucleotide sequences ^b)^ |
| --- | --- | --- |
| rCerPrP_94–233_ | CerPrP94-F | 5’-TTCATATGGGTCAAGGTGGTACCCAC-3’ |
|  | CerPrP-R | 5’-TTGGATCCTCATGCCCCTCTTTGGTAA-3’ |
| rMo^N^–CerPrP | MoPrP-F ^a), b)^ | 5’-TTCATATGAAAAAGCGGCCAAAGCCTG-3’ |
|  | Mo-CerPrP-R ^a)^ | 5’-GGGGTACGGTACATGTTTTCACG-3’ |
|  | Cer-MoPrP-F ^a)^ | 5’-CGTTACCCCAACCAAGTGACT-3’ |
|  | CerPrP-R ^a), b)^ | 5’-TTGGATCCTCATGCCCCTCTTTGGTAA-3’ |
| rCer^N^–MoPrP | CerPrP-F ^a), b)^ | 5’-TTCATATGAAGAAGCGACCAAAACCTG-3’ |
|  | Mo-CerPrP-R ^a)^ | 5’-GGGGTACGGTACATGTTTTCACG-3’ |
|  | Cer-MoPrP-F ^a)^ | 5’-CGTTACCCCAACCAAGTGACT-3’ |
|  | MoPrP-R ^a), b)^ | 5’-TTGGATCCTAGGATCTTCTCCCGTCGTAATA-3’ |
| rCer–Mo^C^PrP | CerPrP-F ^c), d)^ | 5’-TTCATATGAAGAAGCGACCAAAACCTG-3’ |
|  | MoPrP^C^-R ^c)^ | 5’-GTCGTAATAAGCCTGGGATTCTTTCTGGTAC-3’ |
|  | MoPrP-R ^d)^ | 5’-TTGGATCCTAGGATCTTCTCCCGTCGTAATA-3’ |
| rMo–Cer^C^PrP | MoPrP-F ^c), d)^ | 5’-TTCATATGAAAAAGCGGCCAAAGCCTG-3’ |
|  | CerPrP^C^-R ^c)^ | 5’- TTGGTAATAGGCCTGGGACTCCCTCTGGTAC -3’ |
|  | rCerPrP-R ^d)^ | 5’-TTGGATCCTCATGCCCCTCTTTGGTAA-3’ |
| rCerPrP–173S_Mo_/177N_Mo_ | CerPrP-F ^a), b)^ | 5’-TTCATATGAAGAAGCGACCAAAACCTG-3’ |
|  | CerN173ST177N-F ^a)^ | 5’-AGCAACCAGAACAACTTTGTGCATGACTG-3’ |
|  | CerN173ST177N-R ^a)^ | 5’-GTTGTTCTGGTTGCTATACTGATCCACT-3’ |
|  | CerPrP-R ^a), b)^ | 5’-TTGGATCCTCATGCCCCTCTTTGGTAA-3’ |
| rMoPrP–169N_Cer_/173T_Cer_ | MoPrP-F ^a), b)^ | 5’-TTCATATGAAAAAGCGGCCAAAGCCTG-3’ |
|  | MoS169NN173T-F ^a)^ | 5’-AATAACCAGAACACCTTCGTGCACGACTGC-3’ |
|  | MoS169NN173T-R ^a)^ | 5’-GGTGTTCTGGTTATTGTACTGATCCACT-3’ |
|  | MoPrP-R ^a), b)^ | 5’-TTGGATCCTAGGATCTTCTCCCGTCGTAATA-3’ |

^a^ Primers used for the amplifying the gene fragments in the first step of the assembly PCR.

^b^ Primers for assembling gene fragments in the second step of the assembly PCR.

^c^ Primes used for the first step reaction to replace the C-terminus of rPrP.

^d^ Primers used for the addition of BamHI site for cloning into pET11a in the second step reaction to replace the C-terminus of rPrP. GGTACC, BamHI site; CATATG, NdeI site

**Supplementary Figure 1.** Presence of proteinase-resistant PrP in the brain homogenates. The 250 μL of 2% BHs of CWD (W), H-BSE (H), L-BSE (L), and C-BSE (C) infected animals (INF), and those of uninfected deer (De) and cattle (Ca) (UNINF), were digested with 40 μg/mL proteinase K (PK) at 37˚C for 30 min in the final reaction volume of 500 μL. PK-digested samples (250 μg brain tissue equivalent) were applied to each well. Immunoreaction was performed using monoclonal antibody T2 directly conjugated with a horse radish peroxidase [[1](#_ENREF_1)]. Molecular weight markers are shown on the left in kDa.

**Supplementary reference**

1. **Shimizu Y, Kaku-Ushiki Y, Iwamaru Y, Muramoto T, Kitamoto T et al.** A novel anti-prion protein monoclonal antibody and its single-chain fragment variable derivative with ability to inhibit abnormal prion protein accumulation in cultured cells. *Microbiology and immunology* 2010;54(2):112-121.
